# Pediatric Tracheostomy: A Quality-of-Life Assessment Study in Saudi Arabia

**DOI:** 10.7759/cureus.47235

**Published:** 2023-10-17

**Authors:** Moath Al-Faleh, Hussain Alsheef, Omar Al-Anzi, Raghad Jabaan, Sarah M AlQahtani, Marwah Alabdulmhsin

**Affiliations:** 1 ENT, King Fahad Specialist Hospital, Dammam, SAU; 2 Pediatric ENT, Maternity and Children Hospital, Dammam, SAU; 3 Emergency Medicine, College of Medicine, Imam Abdulrahman Bin Faisal University, Dammam, SAU

**Keywords:** quality of life (qol), caregiver, home care, pediatric tracheotomy health status instrument, airway, saudi arabia, pthsi, pediatric tracheostomy

## Abstract

Introduction: The pediatric population undergoes tracheostomy for a variety of reasons. For a child and their family, having a tracheostomy means learning a new way of life and facing several social, psychological, medical, and economic challenges. Our analysis of the literature indicates that this is the first study of its kind, using the Pediatric Tracheotomy Health Status Instrument (PTHSI) tool to assess the quality of life (QoL) following tracheostomy in pediatric patients and their caregivers in the Kingdom of Saudi Arabia (KSA).

Methods: This was a descriptive cross-sectional study for tracheostomized children's QoL evaluation. The medical records of the Maternity and Children Hospital, Dammam, KSA, were used to identify the patients and their caregivers. A higher score on the validated PTHSI indicated a better result.

Results: From a total of 56 patients, 24 were included in this study. Based on the PTHSI tool, the overall mean score was 93.3/150 (62.28%) and this indicated a good QoL score. Analysis of the correlation between the PTHSI score and other variables indicated no association between the total PTHSI score and the age or gender of the patient or the duration of the tracheostomy (p-value > 0.05). However, we found families of children with major medical comorbidities had lower scores (p-value = 0.03) and their QoL was affected much more than families of patients who did not have major medical comorbidities.

Conclusion: Tracheostomy care for pediatric patients can significantly affect the QoL of patients and their families. Our findings using the validated PTHSI tool showed poorer QoL compared to other studies, suggesting the need for future home care training programs to support tracheostomized children and their families, particularly those with comorbidities, who tend to have lower QoL scores and require more organized support.

## Introduction

The pediatric population undergoes tracheostomy for a variety of reasons, like chronic lung disease, genetic conditions, and upper airway abnormality [1]. Tracheostomy care is carried out at home by parents for most of the children who undergo tracheostomies, after discharge [1].

It is challenging to prepare a house for the stay and care of a tracheostomized child. Feeding schedules and home nursing assistance must be established, long-lasting medical equipment must be obtained, and caregivers must be taught how to care for a tracheostomy at home [1,2]. The latter includes not just daily care (such as suctioning and replacing the tracheostomy tube), but also knowing who to contact for tracheostomy care, when to contact them, and what to do if and when an issue arises (such as bleeding or inadvertent decannulation) [1]. Home routines are modified to meet the requirements of the entire family, not just the tracheostomized child, necessitating thorough training and instruction prior to hospital discharge [1]. For the child and their family, having a tracheostomy means learning a new way of life. Patients with a tracheostomy face several social, psychological, medical, and economic challenges [2]. 

There is significant distress among caregivers of children with tracheostomies, according to existing quality of life (QoL) studies [3], as well as a detrimental influence on their mental health [4]. Furthermore, significant tracheostomy-related adverse events such as airway obstruction, decannulation, hemorrhage, and infection are common in modern world centers [5], with morbidity rates ranging from 24% to 58% [6]. Data from superior facilities, however, has shown that even for many of the highly educated and technologically savvy responders, the transfer to home care proved to be a difficult issue [7].

Half of the parents caring for young children in a United Kingdom (UK) research study felt they required support on a daily basis [8]. According to a North American study, 64% of parents providing tracheostomy care had a household member with an undergraduate, graduate, or Ph.D. degree, 59% received daily home nursing care, and 11% got 24-hour home nursing [9]. Regardless of this, 3% of patients went back to the hospital during the first 24 hours of their discharge, 15% visited an emergency room or urgent therapy facility within a week, and 38% required urgent treatment for tracheostomy-related issues within a month [9]. 

To the very best of our knowledge, and in light of our examination of the literature, this is the first study done using the Pediatric Tracheotomy Health Status Instrument (PTHSI) tool to assess the QoL for pediatric tracheostomy and their caregivers in the Eastern province of the Kingdom of Saudi Arabia (KSA). In our healthcare institutions, we lack well-established home care training programs for pediatric tracheostomy and procedures on how to identify and respond to medical emergencies, which puts immense stress on the caregivers, affecting their daily activities and their QoL. Thus, the goal of this study is to assess the QoL of patients and families providing care for children with tracheostomies to establish a database for future home care training programs for pediatric tracheostomy and to establish support for all tracheostomized children and their families.

## Materials and methods

The study included a descriptive cross-sectional evaluation of the QoL for children with tracheostomies. The medical data of the Maternity and Children Hospital, Dammam, KSA, were used to identify pediatric patients and their carers. There were 56 patients in the pediatric age group (≤ 16 years), both male and female, who underwent the tracheostomy procedure and follow-up at Maternity and Children Hospital between September 2017 and May 2022. Of these, 32 were excluded due to loss in follow-ups. English and Arabic speaking families were included. The study was approved by the Scientific and Clinical Studies and Research Department of Maternity and Children Hospital, Dammam, KSA (approval number: PS-2022-001).

Data were collected via phone interviews with caregivers using the PTHSI in Arabic language, which was translated with forward and backward translation and checked. The PTHSI tool is validated as a QoL questionnaire [9]. It focuses on four domains, namely: 1. physical symptoms, 2. frequency and impact of medical visits, 3. QoL of the child from the caregiver's perspective, and 4. QOL of the caregiver [9]. During the psychometric evaluation and approval, the assignment of these domains was reviewed and determined to be appropriate. Each domain had items that were consistent. Scores on the PTHSI range from 0 to 150. A greater overall QoL is indicated by higher ratings [9]. No modifications were made to the PTHSI instrument. There are seven items for the physical symptoms domain, three items for the frequency of medical visits domain, three items for the QoL of the child domain, and 17 items for the caregiver burden domain; hence, a total of 30 items.

Bias was minimized as we took all patients on our active registry who underwent tracheostomy and had routine follow-up. They were interviewed via phone by a physician after formal consent was obtained over the phone. The questionnaire was subsequently completed by the child’s caregiver over the phone. An Arabic interpretation was used for translation of the questions when needed, every single question was explained to the caregiver in both English and Arabic languages, and all related questions were answered. Assistance was provided when needed to complete the survey.

All data were analyzed using the IBM SPSS Statistics for Windows, Version 25.0 (Released 2017; IBM Corp., Armonk, New York, United States). The scores of all of the PTHSI domains were added to create the final score. The association between the child's attributes and the overall PTHSI score was evaluated using Spearman's correlation. The standard for determining statistical significance was p ≤ 0.05.

## Results

**Figure 1:**
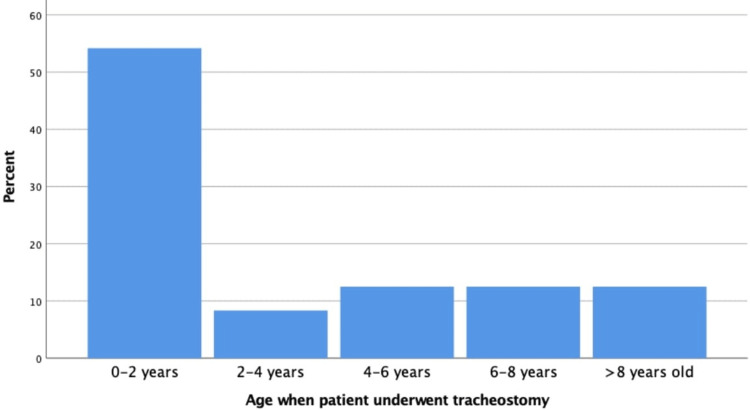
Distribution of patients according to the age at which they underwent tracheostomy

**Figure 2:**
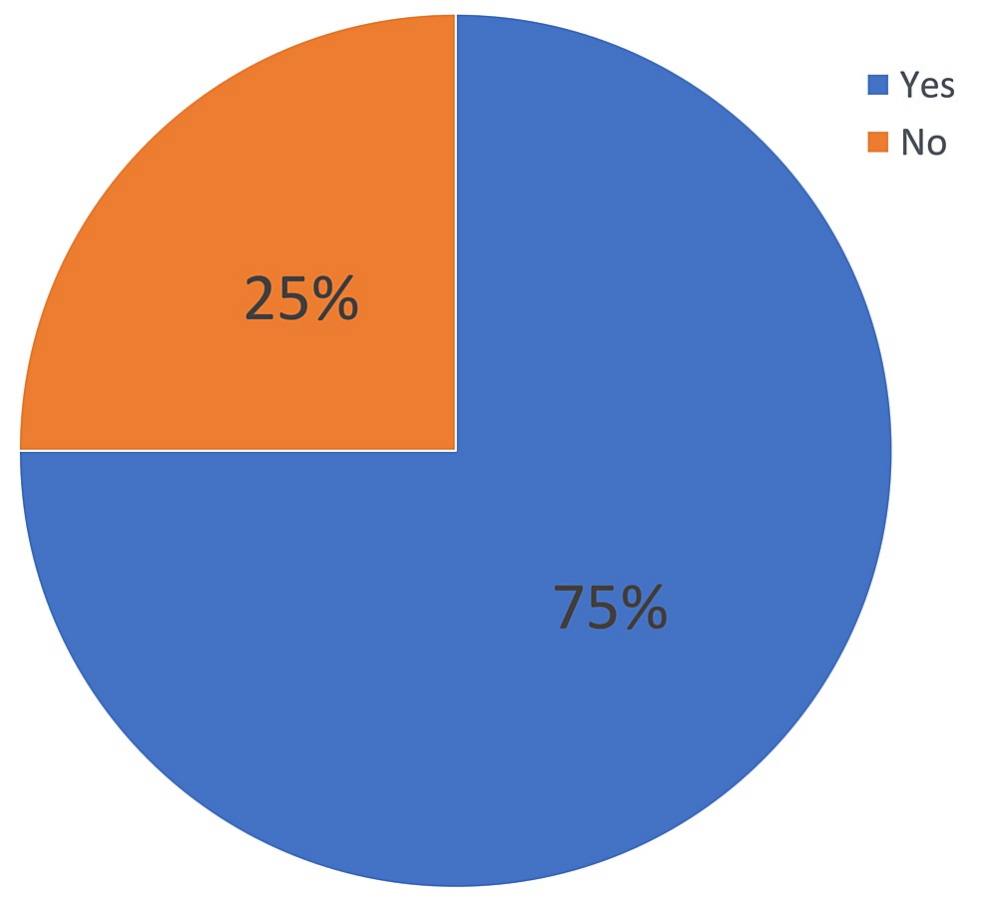
Distribution of major medical conditions among the participants

A total of 24 children were included in the study of whom 15 (62.5%) were males and nine (37.5%) were females (Table 1). The mean age in months was 79 months, the youngest participant was 12 months while the eldest one was 180 months old. A total of 13 (62.5%) participants had their tracheostomies performed while under two years of age, two (8.3%) were aged two to four years, three (12.5%) were aged four to six years, three (12.5%) were aged six to eight years, and three (12.5%) underwent tracheostomy while they are older than eight years old (Figure 1). Major medical conditions (cardiac, neurological, pulmonary) among our participants was present in 18 (75%) patients (Figure 2).

**Table 1:** Gender distribution of the participants

Gender	Frequency	Percent
Female	9	37.5
Male	15	62.5
Total	24	100.0

Correlation between PTHSI score and other variables

The overall mean PTHSI score was 93.3/150 (62.28%), which indicated a good QoL (Table 2). In determining correlation between PTHSI score and other variables, there was no significant correlation between total PTHSI score and duration of tracheostomy, age, or gender (p-value > 0.05). However, we found families of children with major medical comorbidities (Figure 2) had lower scores (p-value = 0.03) and their QoL was affected much more than families of patients who did not have major medical comorbidities.

**Table 2:** Scores of each category of PTHSI along with the total score PTHSI: Pediatric Tracheotomy Health Status Instrument

Category	Minimum	Maximum	Mean Score
Physical Symptoms	8	30	19.8
Medical Visits	6	30	21.9
Quality of Life	0	16	3.5
Stress & Coping	21	69	41.0
Total (out of 150)	57	140	93.3

## Discussion

Generic QoL measurements are less therapeutically relevant than disease-specific QoL measures [10]. Generic measurements, on the other hand, may be used to compare the burden of QoL and treatment benefits across conditions [11]. Both metrics are included in the PTHSI. According to the authors, PTHSI was appropriate for this study because it could give both measures in an integrated manner for a disease-specific condition (having a tracheostomy).

Evidence from more developed centers has reported that the transition to homecare proved a daunting challenge even for many of the highly educated and computer-literate respondents [7], who found the care of a tracheostomy patient complicated and intimidating. In the current study, the overall mean score was 93.3/150 (62.28%), which is good. However, it still does not compare favorably to other studies done at Boston Children’s Hospital and Cape Town Red Cross War Memorial Children’s Hospital using the same PTHSI questionnaire [9,2]. The children in our study reported less favorable scores in the domains of frequency of physical symptoms (19.8 compared to 20.6 in Boston and 24.8 in Cape Town), QoL of the caregiver (41.0 compared to 48.9 in Boston and 62.3 in Cape Town), and QoL of the child, which was the worst, (3.5 compared to 8.7 in Boston and 8.8 in Cape Town) [9,2]. The overall mean score in the current study (93.3/150, 62.28%) was lower than the overall PTHSI mean score of the study in Cape Town Red Cross War Memorial Children’s Hospital (110.2/150, 73.5%).

Major medical comorbidities in children resulted in significantly lower overall PTHSI scores. It is well known that families in this situation face numerous additional stressors. Such tracheostomized patients could also be less agreeable when receiving tracheostomy care, and thus it's clear that this subset of patients and their families need more specialized care. For these children and their families, a thorough tracheostomy care regimen would be most beneficial. Like in our study, the majority of tracheostomized children have significant comorbidities in other studies too [2,9]. Therefore, before discharging these patients home, programs must risk-stratify this subgroup of patients so that additional supportive measures may be put in place.

Common complications in patients with tracheostomies include tube obstruction, mucous plug or foreign body aspiration, and tube dislodgement. An improved prognosis and a more effective treatment plan can result from a home care provider's prompt recognition of symptoms and signs. Common symptoms include respiratory distress, frequent cough, wheezy chest, and cyanosis [12]. This study therefore highlights the stress that these families can experience when caring for tracheostomized patients, as well as how this can negatively impact the QoL of the patients and their families. It also emphasizes the significance of thorough in-hospital training and social support, which can effectively provide families with the required tracheostomy caregiving skills, including coping with emergencies, which can help reduce levels of stress and improve the QoL of both the patient and their family.

Having a well-established pediatric otolaryngology program will aid in providing evidence-based practice to reduce the risk of complications and provide support to families caring for children with tracheostomies [13-15].

The limitation of this study is the relatively small participant number, primarily due to the loss during follow-up, potentially limiting the generalizability of the findings. The study was conducted in a single healthcare institution, which might limit the external validity of the findings to other populations. The results may not reflect the broader diversity of patient characteristics and care practices observed in multi-center or community-based studies.

## Conclusions

To the best of our knowledge, this is the first study done using the PTHSI tool to assess the QoL of pediatric tracheostomy patients and their caregivers in the Eastern province of KSA. Caring for pediatric tracheostomy patients can significantly affect the QoL of the families too. Using data collected using the PTHSI tool, it was seen that QoL was lower compared to the other studies. There is a need to establish home care training programs to support all tracheostomized children and their families, especially as children with comorbidities tend to have lower QoL ratings and need more organized support.
